# Multi-omics integration identifies diverse transcripts and chromatin accessibility profiles in *Monopterus albus* gonads

**DOI:** 10.1038/s41597-025-06478-4

**Published:** 2025-12-27

**Authors:** Yangyang Li, Yuanli Zhao, Hongrui Luo, Kaifeng Meng, Jiarui Song, Jiayi Yang, Wei Hu, Daji Luo

**Affiliations:** 1 Department of Obstetrics and Gynecology, Center for Reproductive Medicine, the Fourth Affiliated Hospital of School of Medicine, and International School of Medicine, International Institutes of Medicine, Zhejiang University, Yiwu, 32200 China; 2 Key Laboratory of Breeding Biotechnology and Sustainable Aquaculture, Institute of Hydrobiology, Hubei Hongshan Laboratory, Chinese Academy of Sciences, Wuhan, 430072 China; 3 College of Fisheries, Guangdong Ocean University, Zhanjiang, 524088 China; 4 College of Advanced Agricultural Sciences, University of Chinese Academy of Sciences, Beijing, 100049 China; 5 State Key Laboratory of Virology, College of Life Sciences, Wuhan University, Wuhan, 430072 China

**Keywords:** Genome-wide association studies, Transcriptomics

## Abstract

The swamp eel (*Monopterus albus*) is an economically important freshwater fish. However, its natural sex reversal contributes to low egg production and fry shortages in aquaculture. The molecular mechanisms governing this process remain poorly understood, hindered by incomplete gene annotations and a lack of comprehensive multi-omics characterization of the gonad. To address this, we performed integrated Iso-seq, RNA-seq, and ATAC-seq profiling across nine key stages of natural female-to-male sex reversal. This approach revealed an extensive, previously unannotated transcriptome landscape, identifying 37,911 novel transcripts. These included 16,900 novel genes and alternative isoforms derived from 24,193 annotated genes. Furthermore, chromatin accessibility mapping identified 2,174 putative cis-regulatory elements, uncovering dynamic regulatory shifts correlated with gene expression changes during sex reversal. Our integrated multi-omics analysis delivers a significantly enhanced transcript annotation framework for *M. albus*, resolving precise polyadenylation sites and surpassing the current NCBI annotation in comprehensiveness. Collectively, this work establishes an essential resource for investigating gonadal development and deciphering sex reversal mechanisms in fish.

## Background & Summary

The swamp eel (*Monopterus albus*) is a freshwater fish widely distributed across East, South, and Southeast Asia, with significant populations in China^1,2^. Valued for its desirable taste and high nutritional content, *M. albus* has emerged as a famous aquaculture species in China, where annual production exceeds 0.3 million tons^2–4^. Beyond its economic significance, it serves as exemplary model organism for studying natural female-to-male sex reversal^5–8^. Individuals typically mature first as females, and following ovarian maturation and ovulation, they undergo a transitional intersex stage before transforming into functional males^9^. This reproductive strategy constrains broodstock availability: female fecundity is limited by small body size, while delayed male maturation reduces spawning capacity. This leads to embryo and fry shortages, hindering *M. albus* commercial production. Gonadal transformation from ovary to testis involves complex transcriptional reprogramming and dynamic chromatin regulation within the *M. albus* genome. However, comprehensive multi-omics characterization—particularly integrated transcriptome and chromatin accessibility profiling across sex reversal—remains limited. This dataset gap impedes elucidation of the molecular mechanisms governing sex reversal and hinders targeted breeding advancements for this species.

Current *Monopterus albus* genome annotations in NCBI and Ensembl rely primarily on *in silico* predictions and limited short-read RNA-seq data^3,10,11^. Critically, short-read sequencing fails to resolve full-length transcript structures due to inherent read-length constraints, yielding fragmented assemblies and inaccurate isoform annotations^3,12^. Long-read isoform sequencing (Iso-seq) overcomes this limitation by capturing complete, unambiguously assembled transcripts. Its successful application across diverse species has revolutionized transcriptome annotation by revealing novel isoforms and precise transcript boundaries^13–15^. Integrating Iso-seq with RNA-seq is thus essential to comprehensively decode the transcriptomic complexity of *M. albus*. Moreover, chromatin accessibility dynamics, measurable via ATAC-seq, directly reflect transcriptional regulatory states^16^. Mapping these changes in gonadal tissue throughout sex reversal provides indispensable insights into *cis*-regulatory logic. Therefore, the synergistic integration of Iso-seq (full-length transcripts), RNA-seq (expression quantitation), and ATAC-seq (*cis*-regulatory landscapes) establishes an unprecedented multi-omics framework and resource to dissect the interplay between transcriptional and epigenetic regulation governing gonadal transformation in *M. albus*.

**Fig. 1:**
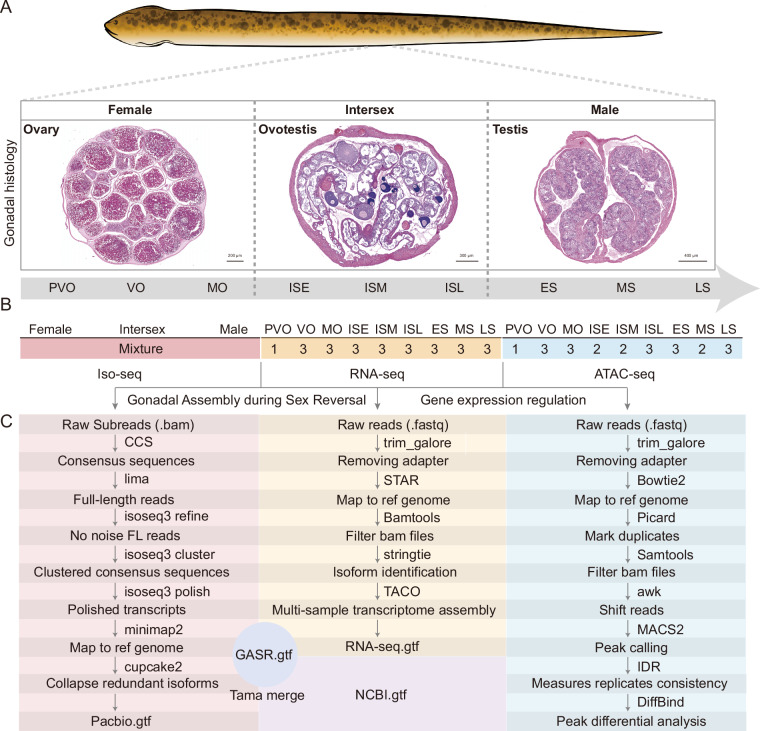
Schematic diagram of gene annotation generation and gene-CREs linkages prediction. (**A**) Samples were obtained from the gonads across the entire sex reversal process: PVO, VO, MO, ISE, ISM, ISL, ES, MS, and LS. Representative histological sections of gonads at female, intersex, and male stages are shown to illustrate morphological characteristics. (**B**) Sequencing strategies for Iso-seq, RNA-seq, and ATAC-seq of the gonads during sex reversal in *M. albus*. “Mixture” indicates that gonadal tissues during sex reversal were pooled into a single sample for Iso-seq. The numbers above the yellow boxes indicate the number of biological replicates per stage for RNA-seq across the nine gonadal developmental stages. The numbers above the blue boxes indicate the number of biological replicates per stage for ATAC-seq across the same nine stages. (**C**) The analysis pipeline of Iso-seq, RNA-seq and merging of assemblies from different sources. Investigation of gene expression regulation through RNA-seq and ATAC-seq.

In this study, we collected gonadal tissues across nine critical stages during sex reversal in *M. albus*: pre-vitellogenic oocytes (PVO), vitellogenic oocytes (VO), mature oocytes (MO), intersexual early (ISE), intersexual middle (ISM), intersexual late (ISL), early-spermatogenic (ES), mid-spermatogenic (MS), and late-spermatogenic (LS). The female phase of gonadal development comprises the PVO, VO, and MO stages (Fig. 1A). In the PVO stage, ovaries primarily contain chromatin-nucleolar and perinucleolar oocytes. The VO stage is characterized by the onset of vitellogenesis, marked by yolk granule deposition and their subsequent fusion into yolk globules and masses. The MO stage exhibits fully mature oocytes with complete yolk aggregation.

During the intersexual phase (ISE, ISM, ISL), oocytes undergo apoptosis while spermatogenesis initiates, resulting in an ovotestis morphology (Fig. 1A). The ISE stage features enlarged gonadal lamellae and proliferation of the germinal epithelium, accompanied by limited spermatogonia and oocytes at various stages of degeneration. In ISM, gonadal lamellae broaden further, with increased spermatogonial presence and only occasional previtellogenic and vitellogenic oocytes. The ISL stage is distinguished by the formation of testicular lobules within the lamellae, containing spermatocytes and rare previtellogenic oocytes.

Testicular maturation proceeds through the ES, MS, and LS stages (Fig. 1A). ES gonads show small lobules with numerous spermatocysts, consisting mainly of spermatogonia and primary spermatocytes. In the MS stage, lumens form within the lobules, and spermatogenesis advances to include both primary and secondary spermatocytes. The LS stage is defined by lobules that fill the lamellar space, with lumens containing all spermatogenic cell types, predominantly secondary spermatocytes and spermatids.

These samples were subjected to parallel full-length transcriptome (Iso-seq), RNA-seq, and ATAC-seq profiling. Through synergistic integration of long-read isoforms, short-read assemblies, and existing annotations, we generated the Gonadal Assembly during Sex Reversal (GASR) resolving 37,911 previously unannotated isoforms (Fig. 1). Complementarily, genome-wide chromatin accessibility profiling identified 2,174 differentially accessible regions (DARs) with putative cis-regulatory functions. Collectively, this integrated multi-omics atlas establishes an essential foundation for unprecedented transcriptomic complexity and dynamic regulation throughout gonadal transformation in *M. albus*.

## Methods

### Ethics statement

All animal procedures and husbandry practices were approved by the Institutional Animal Care and Use Committee (IACUC) of the Institute of Hydrobiology, Chinese Academy of Sciences (Approval ID: IHB/LL/2023039). The study was conducted in full compliance with the Guiding Principles for the Care and Use of Laboratory Animals and the local guidelines on the ‘Management and Use of Laboratory Animals of Hubei Province’. All efforts were made to minimize animal suffering. The fish were housed under optimal conditions, with professional staff providing feeding and care. No endangered or protected species were involved.

### Sample preparation

Female, intersex, and male *M. albus* used for this study were obtained from Haoxiang Fish Farm in Wuhan City, Hubei Province, China. These fish were caught in the wild during the breeding season, which typically occurs from May to August in Wuhan, China. After one week of temporary acclimation, the fish were euthanized by immersion in buffered MS222 solution (500 mg/L; E10521, Sigma-Aldrich). Gonadal tissues of each individual were dissected and divided into four subsamples. One subsample was used for hematoxylin and eosin (H&E) staining to determine the developmental stage of the gonad. Two additional subsamples were used for the construction of RNA-seq and ATAC-seq libraries, respectively. The final subsample, consisting of a mixture of gonadal tissues from all nine stages, was pooled to construct the Iso-Seq library.

### Library construction for RNA-seq

Total RNA served as the initial material for library preparation. Messenger RNA was isolated utilizing poly-T oligo-attached magnetic beads. Fragmentation was conducted with divalent cations at elevated temperatures in First Strand Synthesis Reaction Buffer (5X). The synthesis of first-strand cDNA was carried out using random hexamer primers and M-MuLV Reverse Transcriptase. Second-strand cDNA synthesis employed DNA Polymerase I and RNase H. After adenylation at the 3′ ends, hairpin loop adaptors were ligated. cDNA fragments between 370–420 bp were purified using the AMPure XP system (Beckman Coulter, Beverly, USA). Sequencing was carried out on the Illumina NovaSeq platform, generating 150 bp paired-end reads.

### Library construction for PacBio Iso-seq

Mixtures (female, intersex, and male) were used to construct an Iso-seq library. The mixed RNA sample was reverse transcribed using the SMARTer PCR cDNA Synthesis Kit. The resulting cDNA underwent amplification, and fragments ranging from 0.5 to 10 kb were selected for subsequent library preparation. Finally, SMRTbell libraries were constructed using the Pacific Biosciences DNA Template Prep Kit 2.0 and sequenced on the PacBio Sequel platform.

### Library construction for ATAC-seq

ATAC-seq was carried out following established protocols. In summary, nuclei were isolated from the samples, and the resulting pellet was resuspended in a Tn5 transposase reaction mixture. The transposition process was incubated at 37 °C for 30 minutes. Afterward, equimolar amounts of Adapter 1 and Adapter 2 were introduced, followed by PCR amplification of the library. Post-PCR, the libraries were purified using AMPure beads, and quality was evaluated with Qubit. Sequencing was completed on the Illumina NovaSeq platform, generating 150 bp paired-end reads.

### Processing of RNA-seq data

Data quality of RNA-seq data was assessed using FastQC (http://www.bioinformatics.babraham.ac.uk/projects/fastqc/), and Trim Galore (http://www.bioinformatics.babraham.ac.uk/projects/trim_galore/) was used to remove adapters and low-quality bases in raw reads. Filtered reads were aligned to the *M. albus* genome (M_albus_1.0) using the STAR aligner with the two-pass mapping mode^17^. The uniquely mapped reads were kept for further analysis. Gene expression matrices were generated using the featureCounts program^18^. Differential gene expression analysis was performed by DESeq2^19^, genes with fold change > 2 and adjusted *p*-value < 0.01 were defined as differentially expressed genes (DEGs).

### Processing of PacBio full-length Iso-seq

We employed multiple programs from PacBio SMRT Analysis, following the guidelines outlined in the SMRT Tools Reference Guide. Consensus sequences were generated from raw subread data using CCS and full-length reads were constructed using lima by primer removal and demultiplexing. Full-Length Non-Chimeric transcripts were identified using Isoseq3 refine by removing polyA and concatemers from full-length reads. Full-Length Non-Chimeric reads were then clustered and polished using the cluster and polish subcommands of Isoseq3, respectively. Specialized long-read aligners minimap2^20^ were selected to map the polished transcripts to the *M. albus* genome (M_albus_1.0).

### Merging of assemblies from RNA-seq and Iso-seq data

Short-read assembly, long-read assembly, and NCBI reference annotations were combined to reassemble the transcriptome of *M. albus*. For short-read RNA-seq, multi-sample assembly was performed by StringTie^21^ and a consensus merged transcriptome was generated by TACO^22^. For Iso-seq, we used Cupcake (https://github.com/Magdoll/cDNA_Cupcake) subcommand collapse_isoforms_by_sam.py to collapse redundant isoforms and filter away 5′ degraded isoforms by filter_away_subset.py. Assemblies were merged by the tama_merge.py script of TAMA^23^. Finally, GASR was compared to the NCBI annotation using SQANTI3 and GffCompare.

### Verification of the polyA site location

Transcripts classified as internal primer transcripts were excluded if the 20-nucleotide sequence following the polyA region contained over 15 adenines. Isoforms that remained after this filtering step were designated as those assembled from long reads. To construct the final transcript model, these long-read isoforms served as the primary dataset, complemented by short-read-assembled isoforms that did not overlap on the same strand. The reference utilized for this analysis was based on the M_albus_1.0 assembly and its corresponding annotation, GCF_001952655.1_M_albus_1.0_genomic.gff^24^.

Based on the GASR isoforms, the 200nt sequence extraction module utilizes the get_polya_sequences function to extract sequence lists of 200 nucleotides in length; The signal frequency analysis module for polyA-related motifs employs the get_motif_frequency function to perform sliding window detection (window size: 200nt) of two polyA-related motifs (AAUAAA and AUUAAA), calculating the percentage frequency of motif occurrences at each nucleotide position; The APA site spacing calculation module uses the load_apa_distance function to determine the distances between alternative polyadenylation (APA) sites within genes and counts the number of APA events with spacing between 100–10,000 nucleotides.

### Processing of ATAC-seq data

Adapter sequences of ATAC-seq reads were trimmed by trim_galore. Trimmed reads were aligned to the *M. albus* genome by Bowtie2. Duplicate reads were filtered out by Picard MarkDuplicates (http://broadinstitute.github.io/picard/) and reads mapped to mitochondria were also removed. Filtered reads were shifted +4 bp for positive strand and −5 bp for negative strand, and shifted reads were applied to MACS2^25^ for peak calling. Normalized tracks were generated by bamCoverage in deepTools with the parameters: --binSize 10 --normalizeUsing RPKM –extendReads. To identify DARs between different stages, ATAC peaks were processed with DiffBind^26^. DARs were identified with a cutoff of FDR <0.01.

## Data Records

All raw RNA-seq reads of the *M. albus* have been submitted to the Sequence Read Archive (SRA) at the National Center for Biotechnology Information under accession numbers SRP578156^27^. Additionally, the complete set of raw full-length Iso-seq reads for this species has been archived in the same database under accession number SRP583726^28^. All raw ATAC-seq data were deposited in the SRA under accession number SRP584005^29^. All datasets are jointly organized under the GEO SuperSeries accession GSE312266^30^. The transcriptome assembly produced in this study has been submitted to the NCBI Transcriptome Shotgun Assembly (TSA) database under accession number GLKV00000000.1^31^. The NCBI and GASR isoforms, as well as Table S1 containing putative gene-CREs linkages during sex reversal, can be accessed through Zenodo under 10.5281/zenodo.17470351^32^.

## Technical Validation

### Quality control of sequencing data

**Fig. 2:**
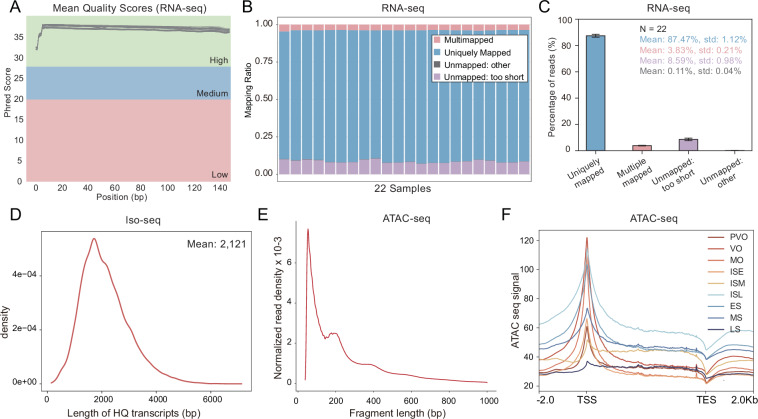
Quality summary of RNA-seq data. (**A**) The mean quality value across each base position in the read. (**B,C**) Summary of mapping ratio for RNA-seq. Uniquely mapped: Reads successfully aligned to a single, specific location in the genome; Multiple mapped: Reads successfully mapped to more than one genomic locations; Unmapped (too short): Reads that could not be aligned because their length was insufficient for reliable mapping; Unmapped (other): Reads that failed to align due to various other issues. (**D**) The length distribution of the high-quality transcripts for Iso-seq. (**E**) Fragment size distribution plot showing enrichment around nucleosome-free and mono-nucleosome-bound fragments. (**F**) Chromatin accessibility around transcription start sites (TSS) and transcription end sites (TES) of 9 developmental stages.

For RNA-seq, the majority of reads had a mean base quality higher than Q30 (Fig. 2A), indicating high sequencing accuracy and reliable data quality for downstream analysis. Across the 22 RNA-seq samples, the average uniquely mapped ratio was 87.47% (Table 1). On average, 3.83%, 8.59%, and 0.11% of the reads were classified as multimapped, unmapped (too short), or unmapped (other), respectively (Fig. 2B,C). The high proportion of uniquely mapped reads suggests minimal contamination, sequencing artifacts, or adapter contamination, ensuring that most reads correspond to actual transcripts from the sample rather than technical noise. For PacBio full-length Iso-seq, a total of 16,408,435 subreads were produced and 559,628 Circular Consensus Sequences (CCS) were generated from subreads. Further refinement using IsoSeq3 identified 449,687 Full-Length Non-Chimeric (FLNC) reads with an average length of 2,040 bp. These FLNC reads represent high-confidence transcript sequences, ensuring accurate isoform reconstruction. After clustering and polishing, 35,340 reads, with an average length of 2,121 bp were deemed suitable for downstream functional annotation (Fig. 2D and Table 2). For ATAC-seq data, the alignment rate ranged from 83.58% to 92.04%, with an average of 87.79% (Table 3). Fragment size distribution plots, showing decreasing and periodical peaks, indicated the nucleosome-free regions (NFR) (<100 bp) and mono-nucleosome-bound regions (~200 bp) (Fig. 2E). ATAC-seq signals around TSSs were significantly higher than other regions (Fig. 2F).

**Table 1:** Summary of alignment results for RNA-seq.

Sample	Input Reads	Mapped Reads	Mapped Ratio%
PVO_rep1	2,12,13,820	1,80,94,110	85.29%
VO_rep1	2,28,37,526	1,98,44,384	86.89%
VO_rep2	2,20,65,387	1,90,07,030	86.14%
VO_rep3	2,18,20,475	1,88,83,301	86.54%
MO_rep1	2,53,56,567	2,23,55,222	88.16%
MO_rep2	2,39,41,746	2,11,68,210	88.42%
MO_rep3	2,37,13,606	2,09,00,013	88.14%
ISE_rep1	2,61,32,066	2,25,32,579	86.23%
ISE_rep2	2,17,68,412	1,85,89,623	85.40%
ISM_rep1	2,71,24,745	2,39,87,513	88.43%
ISM_rep2	2,82,09,237	2,48,54,341	88.11%
ISL_rep1	2,82,03,702	2,47,58,670	87.79%
ISL_rep2	2,26,55,994	2,01,69,487	89.02%
ISL_rep3	2,64,44,990	2,34,60,576	88.71%
ES_rep1	2,18,70,999	1,93,86,368	88.64%
ES_rep2	2,58,70,534	2,26,86,023	87.69%
ES_rep3	3,32,68,898	2,90,84,151	87.42%
MS_rep1	2,14,44,323	1,84,44,480	86.01%
MS_rep2	2,14,90,134	1,86,66,377	86.86%
LS_rep1	2,19,84,726	1,94,47,974	88.46%
LS_rep2	2,33,86,766	2,06,50,534	88.30%
LS_rep3	2,19,79,872	1,92,90,804	87.77%

**Table 2:** Statistic of Iso-seq data in *M. albus*.

Type	Total bases(bp)	Total reads number	Average length(bp)
Subreads	56 G	1,64,08,435	1894
CCS	42,10,63,698	5,59,628	2209
FL	34,78,26,235	4,60,176	2069
FLNC	33,52,58,838	4,49,687	2040
Cluster and Polished	3,30,08,988	35,340	2121

**Table 3:** Summary of alignment results for ATAC-seq.

Sample	Input Reads	Mapped Reads	Mapped Ratio%
PVO_rep1	13,23,86,050	11,54,20,843	87.19%
VO_rep1	21,67,70,480	19,41,28,215	89.55%
VO_rep2	14,83,95,316	13,19,80,525	88.94%
VO_rep3	23,77,67,200	20,69,59,298	87.04%
MO_rep1	14,64,04,558	13,47,47,138	92.04%
MO_rep2	17,74,30,948	15,78,34,934	88.96%
MO_rep3	18,99,88,298	16,84,48,833	88.66%
ISE_rep1	17,09,68,166	15,30,98,151	89.55%
ISE_rep2	14,81,60,008	13,35,39,281	90.13%
ISE_rep3	16,65,80,562	15,22,36,006	91.39%
ISL_rep1	8,63,12,190	7,45,30,153	86.35%
ISL_rep2	11,05,01,926	9,43,40,359	85.37%
ISL_rep3	16,31,90,610	13,88,88,424	85.11%
ISM_rep1	14,44,47,304	12,28,00,763	85.01%
ISM_rep2	17,19,61,640	15,00,69,164	87.27%
ISM_rep3	11,19,96,850	9,43,11,041	84.21%
ES_rep1	15,68,17,788	14,22,12,672	90.69%
ES_rep2	16,74,70,930	15,02,74,136	89.73%
ES_rep3	10,23,17,872	8,86,99,025	86.69%
MS_rep1	9,90,09,538	8,62,64,497	87.13%
MS_rep2	8,95,74,724	7,90,43,512	88.24%
MS_rep3	9,18,41,234	8,07,79,665	87.96%
LS_rep1	16,99,00,586	14,20,03,533	83.58%
LS_rep2	10,02,32,888	8,63,67,729	86.17%
LS_rep3	17,82,73,748	15,63,64,505	87.71%

### Quality control of annotation GASR

**Fig. 3:**
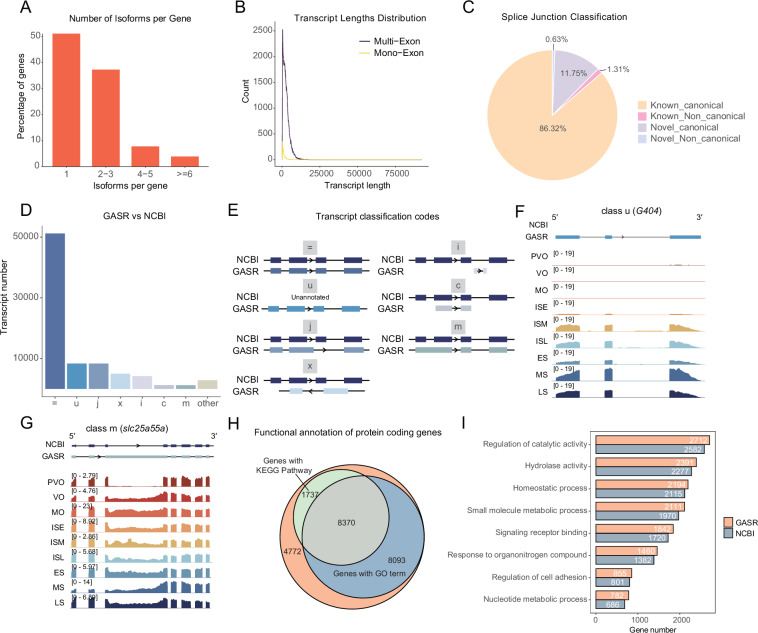
Summary and evaluation of the assembled gene annotation. (**A**) Distribution of isoform numbers per gene in our assembly GASR. (**B**) Mono-exon and multi-exon transcript lengths distribution in GASR. (**C**) Summary of splice junction types identified from GASR. (**D**) Transcript structure comparison between GASR and NCBI reference. (**E**) Explanation of transcript classification codes. =: complete, exact match of intron chain, i: fully contained within a reference intron, u: unknown and intergenic, c: contained in reference, j: multi-exon with at least one junction match, m: retained intron, x: exonic overlap on the opposite strand. (**F,G**) Representative IGV visualization of genes from different transcript classification codes, such as class u and class m. (**H**) Genes annotated with GO terms and KEGG pathways. (**I**) GO term assignment comparison between NCBI reference and GASR.

After processing gonadal RNA-seq and Iso-seq data and performing transcriptome assembly, we integrated the results with the NCBI reference annotation to produce our final annotation version, GASR. GASR contains 41,093 genes and 82,545 transcripts, of which 37,911 were derived from reassembly. Of these, 51.1% of the genes contain multiple transcript isoforms (Fig. 3A), and the majority of transcripts consist of multiple exons (Fig. 3B). Additionally, GASR identified 33,743 novel splice junctions, 32,020 of which follow canonical splicing patterns (Fig. 3C). Structural differences were observed between GASR and NCBI annotation (Fig. 3D,E), such as unknown novel isoforms (class u, Fig. 3F) and retained introns (class m, Fig. 3G). Among the 41,093 annotated genes, 22,972 were classified as protein-coding. Functional annotation revealed that 16,463 genes were mapped to Gene Ontology (GO) terms, providing insights into their biological processes, molecular functions, and cellular components. Furthermore, 10,107 genes were assigned to pathways in the KEGG database (Fig. 3H). For certain GO terms, the number of genes identified in the GASR exceeded that in the NCBI reference annotation, indicating that GASR provides an expanded functional landscape (Fig. 3I).

### Quality control of polyA site location

**Fig. 4:**
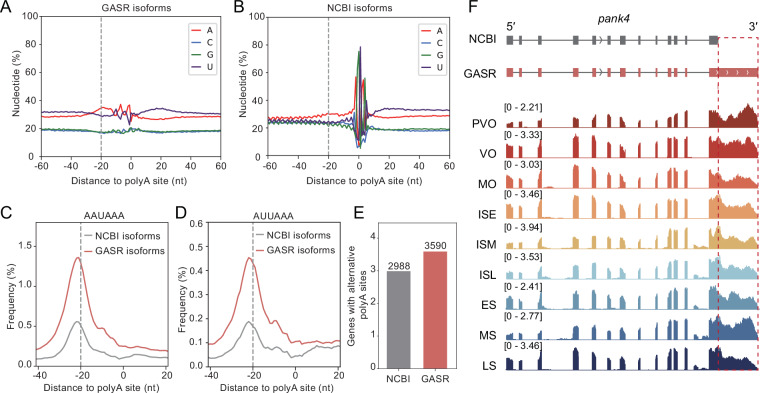
Enhanced polyA site annotation in GASR compared to the NCBI reference. (**A**) Nucleotide distribution around polyA sites in GASR. (**B**) Nucleotide distribution around polyA sites in NCBI. (**C,D**) PolyA processing signal (AAUAAA and AUUAAA) density around PolyA sites based on NCBI and GASR. (**E**) Quantification of genes containing alternative polyA sites in the NCBI reference and GASR. (**F**) IGV visualization of *pank4* RNA-seq signal, where GASR more accurately matched the 3′UTR length than the NCBI reference.

We assessed the 3′ end quality in GASR by analyzing nucleotide composition around predicted polyadenylation (polyA) sites. The results revealed a strong enrichment of adenine (A) in the 20 bases upstream of the polyA site, while the 20 bases downstream were predominantly uracil (U)-rich (Fig. 4A). Compared to the NCBI annotation, the polyA sites identified in GASR exhibited a more balanced AT/CG ratio in the flanking regions (Fig. 4B). In addition, compared to NCBI annotation, GASR displayed a notable enrichment of AAUAAA (Fig. 4C) and AUUAAA (Fig. 4D) sequences within the 20 base pairs upstream of the polyA sites, reflecting a higher frequency of conserved 3′ end processing signals.

To further evaluate the completeness of GASR, we examined genes with multiple polyA sites. The results indicate that GASR includes a significantly higher number of genes with APA compared to the NCBI annotation (Fig. 4E). To validate the accuracy of our polyA site annotations, we displayed the *pank4* gene during sex reversal in *M. albus* (Fig. 4F). Therefore, GASR improved accuracy in identifying polyA sites compared to existing gene annotation.

### Gene-CREs linkages prediction

**Fig. 5:**
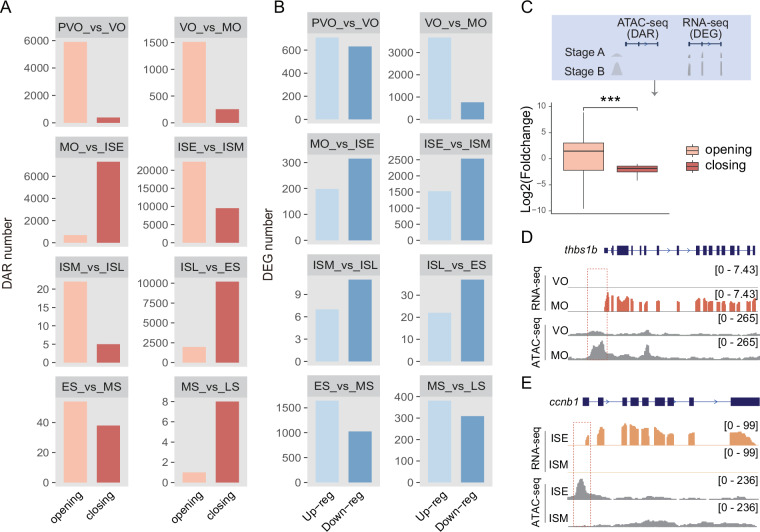
Putative gene-CREs linkages identification. (**A**) The number of DARs with different types at later stages was identified through pairwise comparison across the entire sex reversal process. (**B**) The number of DEGs with different types at later stages was identified through pairwise comparison across the entire sex reversal process. (**C**) Expression changes of the closest DEGs to DARs within 50 kb during gonad development. Statistical differences between groups were analyzed by wilcox.test. ***P < 0.001. (**D,E**) Examples of gene-CREs linkages. Gene expression correlates with chromatin accessibility in the promoter, where regions with altered accessibility act as regulatory elements that regulate gene expression, as demonstrated by *thbs1b* and *ccnb1*.

To provide a data resource for exploring the transcriptional regulation underlying gene expression changes, we utilized ATAC-seq to explore chromatin accessibility during sex reversal. Putative gene-CRE linkages were investigated based on RNA-seq and ATAC-seq data. In total, 60,199 DARs (Fig. 5A and Table 4) and 13,776 DEGs (Fig. 5B and Table 5) were identified by pairwise comparison. For each DAR, we computed correlations between chromatin accessibility and the expression of the closest DEGs to DARs within 50 kb (Fig. 5C). This dataset includes expression changes of DEGs located near both opening and closing DARs, with representative examples such as *thbs1b* (Fig. 5D) and *ccnb1* (Fig. 5E), thus facilitating examination of the relationship between gene expression and chromatin accessibility. Gene-DARs links exhibiting concordant dynamics of chromatin accessibility and gene expression were identified as putative gene-CREs linkages (Table S1^32^). These data further underpinned the cis-regulatory potential of DARs and provided a set of DARs associated with sex reversal.

**Table 4:** Summary of DARs during sex reversal.

Comparison	DAR Type	DAR number
PVO_vs_VO	closing	388
PVO_vs_VO	opening	5902
VO_vs_MO	closing	255
VO_vs_MO	opening	1511
MO_vs_ISE	closing	7318
MO_vs_ISE	opening	689
ISE_vs_ISM	closing	9518
ISE_vs_ISM	opening	22340
ISM_vs_ISL	closing	5
ISM_vs_ISL	opening	22
ISL_vs_ES	closing	10189
ISL_vs_ES	opening	1961
ES_vs_MS	closing	38
ES_vs_MS	opening	54
MS_vs_LS	closing	8
MS_vs_LS	opening	1

**Table 5:** Summary of DEGs during sex reversal.

Comparison	DEG Type	DEG number
PVO_vs_VO	Up-reg	708
PVO_vs_VO	Down-reg	631
VO_vs_MO	Up-reg	3669
VO_vs_MO	Down-reg	762
MO_vs_ISE	Up-reg	198
MO_vs_ISE	Down-reg	315
ISE_vs_ISM	Up-reg	1524
ISE_vs_ISM	Down-reg	2534
ISM_vs_ISL	Up-reg	7
ISM_vs_ISL	Down-reg	11
ISL_vs_ES	Up-reg	22
ISL_vs_ES	Down-reg	37
ES_vs_MS	Up-reg	1643
ES_vs_MS	Down-reg	1025
MS_vs_LS	Up-reg	381
MS_vs_LS	Down-reg	309

## Data Availability

All datasets generated and analyzed during this study have been deposited in publicly accessible repositories. The RNA-seq, Iso-Seq, and ATAC-seq datasets are organized under the GEO SuperSeries GSE312266^30^, and the corresponding raw reads are available in the NCBI Sequence Read Archive (SRA) under the BioProject accession numbers SRP578156^27^, SRP583726^28^, and SRP584005^29^, respectively. The transcriptome assembly generated in this study has been deposited in the NCBI TSA database under accession number GLKV00000000.1^31^. All related data, including the NCBI and GASR isoforms and Table S1 containing putative gene–CRE linkages during sex reversal, are publicly available via Zenodo (10.5281/zenodo.17470351^32^).

## Code availability

All software employed throughout this research is openly accessible, with parameters explicitly outlined within the Methods section. When specific settings were not detailed for any given tool, recommended defaults from the original developers were applied. The code for this paper was deposited at GitHub: https://github.com/liyangyang12/Multi-omics-data-of-Monopterus-albus.
